# Whole genome sequencing of *Klebsiella pneumoniae* clinical isolates sequence type 627 isolated from Egyptian patients

**DOI:** 10.1371/journal.pone.0265884

**Published:** 2022-03-23

**Authors:** Shymaa Enany, Samira Zakeer, Aya A. Diab, Usama Bakry, Ahmed A. Sayed

**Affiliations:** 1 Department of Microbiology and Immunology, Faculty of Pharmacy, Suez Canal University, Ismailia, Egypt; 2 Biomedical Research Department, Armed Force College of Medicine, Cairo, Egypt; 3 Genomic research program, Basic research department, Children’s Cancer Hospital Egypt, Cairo, Egypt; 4 Department of Biochemistry, Faculty of Science, Ain Shams University, Cairo, Egypt; Government College University Faisalabad Pakistan, PAKISTAN

## Abstract

*Klebsiella pneumoniae* is considered a threat to public health especially due to multidrug resistance emergence. It is largely oligoclonal based on multi-locus sequence typing (MLST); in Egypt, ST 627 was recently detected. Despites the global dissemination of this ST, there is still paucity of information about it. Herein, we used 4 *K*. *pneumoniae* ST627 for whole genome sequencing utilizing an Illumina MiSeq platform. Genome sequences were examined for resistance and virulence determinants, capsular types, plasmids, insertion sequences, phage regions, and Clustered Regularly Interspaced Palindromic Repeats (CRISPR) regions using bioinformatic analysis. The molecular characterization revealed 15 and 65 antimicrobial resistance and virulence genes, respectively. Resistance genes such as *tet(D)*, *aph(3’’)-Ib*, *aph(6)-Id*, *blaTEM-234*, *fosA*, and *fosA6*; were mainly responsible for tetracycline, aminoglycoside, and fosfomycin resistance; respectively. The capsular typing revealed that the four strains are KL-24 and O1v1. One plasmid was found in all samples known as pC17KP0052-1 and another plasmid with accession no. NZ_CP032191.1 was found only in K90. IncFIB(K) and IncFII(K) are two replicons found in all samples, while ColRNAI replicon was found only in K90. Entero P88, Salmon SEN5, and Klebsi phiKO2 intact phage regions were identified. All samples harbored CRISPR arrays including CRISPR1 and CRISPR2. Our results shed light on critical tasks of mobile genetic elements in ST 627 in antibiotic resistance spreading.

## Introduction

*Klebsiella pneumoniae* is a non-motile Gram-negative bacterium. It is one of the most opportunistic microorganisms that associated with community-acquired and nosocomial infections and contributed progressively to health care associated infections [1]. *K*. *pneumoniae* is responsible for several human infections including urinary tract, respiratory tract, and bloodstream infections [2]. Spreading of *K*. *pneumoniae* has become a major public health problem specially after the emergence of multi-drug resistant isolates [3]. Many studies have proved that the antimicrobial resistance in *K*. *pneumoniae* is a clear and present danger [3, 4]. *K*. *pneumoniae* is responsible for nearly one third of all the Gram-negative infections [5]. In recent years, WHO has listed *K*. *pneumoniae* as a critical priority microbe due to the high morbidity and mortality accompanied with its infection [6]. Genotyping for such an important pathogen is a demand. Multi-locus sequence typing (MLST) is one of the molecular methods used for characterization of bacterial isolates genetic relationship and it is mainly intended for molecular epidemiology of microbes of public health issues [7, 8]. Using MLST has revealed that *K*. *pneumoniae* is largely oligoclonal; many sequence types have been recorded; ST 11, ST 14, ST 15, ST 26, ST 101, ST 147, ST 149, ST 231, ST 258, ST 627, and ST 977 [9]. Many STs have been specific for certain geographical areas and some have been epidemic and/ or endemic [10]. Inasmuch, ST 258 has disseminated especially in North America, Latin America, and several countries in Europe [9, 11, 12] while ST 11 has been disseminated in Asia and South America [9, 13]. In Egypt, ST 627 was recently detected in four *K*. *pneumoniae* isolates out of six [14]. ST 627 was previously detected in other countries; a two-year epidemiological study in Korea of 362 Enterobacteriaceae strains showed the presence of many sequence types of *K*. *pneumoniae* strains which become endemic in their country [15]. These types showed moderate to high resistance rates to many antimicrobial agents. ST 627 was one of these types, which were carried by Tn4401 [15]. Another conducted one-year study in Greece showed the presence of the same ST between other sequence types of Klebsiella strains which forms a great worse issue in treatment options and infection control practices in health care facilities [16]. This sequence type not only presents in hospitalized patients colonized with Klebsiella, but also it has been found powerfully in pediatric oncology wards in cancer children [17]. This finding was confirmed by the study conducted in Czech Republic that raises the alarm to aware the importance of this sequence type for further studies [17]. Surprisingly, this sequence type is not only found in human beings but also found in poultry; this finding was reported by a Lebanese group in 2016–2017 [18]. Pertinently, *K*. *pneumoniae* isolates were detected in Spain from samples of fresh products of chicken and turkey were harboring ST 627 of high resistance rates to many antibiotics [19]. These findings must draw attention to the possible transfer of this sequence type from animals to human the same as from human to human [19]. Despites the global dissemination of this sequence type, there is still paucity of information on it. A better understanding of the transmission and pathogenesis of *K*. *pneumoniae* ST 627 via genotyping methods is a stipulation. Whole-genome sequencing (WGS) could provide a far superior genomic resolution and full genetic information on the entire bacterial genome covering all relevant genomic characteristics. Beyond bacterial identification and molecular characterization, WGS could present a substantial source which could be availed to foretell the microbe’s phenotype [20].

In this study, we use WGS to understand the genetic variations between clinical isolates of *K*. *pneumoniae* ST 627 isolated from Egyptian patients and to discern their relatedness with another reported genome retrieved from the NCBI database. As well, we aimed to determine the molecular characterization of antibiotic resistance genes, virulence factors, and various mobile genetic elements accompanied with *K*. *pneumoniae* ST 627.

## Results and discussion

**Fig 1:**
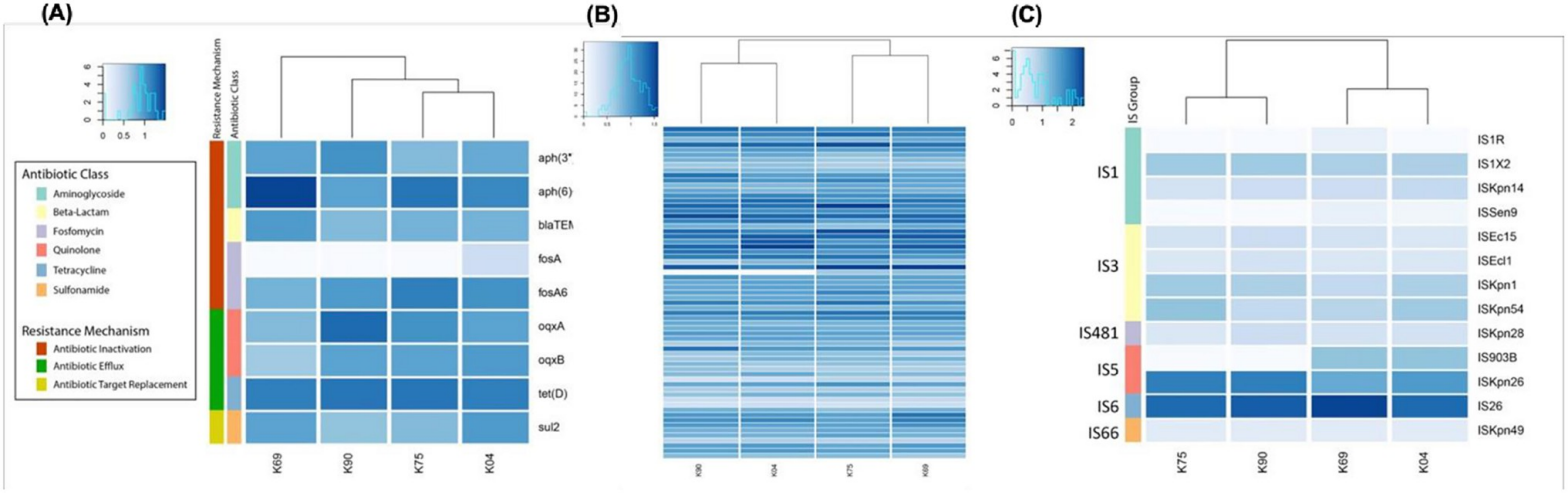
(A): Heatmap shows the relative copy numbers of the resistance genes. Isolates are arranged on the X axis and are given names starting with K, followed by the number of each sample. Genes are plotted on the Y- axis. Genes with a darker blue colour have a relatively high copy number in a given sample, (B): Heatmap shows each virulence factor in each isolate. Isolates are arranged on the X axis, while virulence factors are plotted on the Y- axis. Virulence factors with a darker blue colour have a relatively high copy number in a given sample, while virulence factors with a lighter blue-white colour have a relatively low copy number in comparison to others, (C): Heatmap shows each insertion sequence in each isolate. Isolates are arranged on the X axis, while insertion sequences are plotted on the Y- axis. Insertion sequences with a darker blue colour have a relatively high copy number in a given sample, while insertion sequences with a lighter blue-white colour have a relatively low copy number in comparison to others.

The molecular characterization of the resistance factors associated with *K*. *pneumoniae* ST 627 isolated from Egypt was implemented. The data analysis has indicated the presence of 15 antimicrobial resistance genes (Fig 1(A)) responsible for the resistance of the bacterial strains to different drug classes. Previous WGS for *K*. *pneumoniae* ST 14 has disclosed a comparable number of resistance genes [21]. This is at variance with what was reported before with other ST types of *K*. *pneumoniae*; ST152 and ST17 harboring 48 and 36 resistance genes; respectively [22]. That was explained by Kumar et al. who suggested the presence of enormous motifs of the genetic and phenotypic variance of *K*. *pneumoniae* clinical isolates and attributed that to the possible horizontal gene transfer [23]. The resistant genes identified in our analysis are *aph(3’’)-Ib*, *aph(6)-Id*, *blaTEM-234*, *fosA*, *fosA6*, *oqxA*, *oqxB*, *sul2* and *tet(D)*. These genes are responsible for three resistance mechanisms named antibiotic inactivation, antibiotic efflux, and antibiotic target replacement. The most predominant resistance mechanism identified is the antibiotic inactivation conferred by *aph(3’’)-Ib and aph(6)-Id* which is responsible for aminoglycoside phosphotransferase that has a major role for aminoglycoside resistance [24, 25]. *blaTEM-234*, *fosA*, and *fosA6* are responsible for conferring fosfomycin resistance [26]. The antibiotic efflux is conferred by *tet(D)*, *oqxA*, and *oqxB*. *tet(D)* is responsible for harboring the tetracycline resistance [27]. The antibiotic target replacement is conferred by *sul2*. It is not surprising that these genes were identified significantly in isolates from Egyptian patients, since it was established in Egypt the frequently usage of fosfomycin in combination with aminoglycosides for the management of many respiratory infections like chronic endobronchial infections [28]. Adding to that, the profuse misuse of tetracycline in Egyptian poultry farms that allows the spreading of tetracycline resistance through the poultry litter or droppings containing tetracycline [29]. A detailed profiling of antimicrobial resistance (AMR) genes, drug classes, and the resistance mechanisms were shown in S1 Table. The wide variety of resistance genes and resistance mechanisms detected here suggested that *K*. *pneumoniae* ST 627 clinical samples might be a possible reservoir of resistance genes to other different species [22] and ensured the success of this sequence type to continue evading different classes of antibiotics.

We exploited the fact that *K*. *pneumoniae* is highly pathogenic and investigated the virulence factors associated with *K*. *pneumoniae* ST 627. Virulence factors profiling of the four samples belonged to ST 627 has revealed the presence of 65 virulence factors (Fig 1(B) and S2 Table). The *fim* genes cluster (*fimA*, *fimB*, *fimC*, *fimD*, *fimE*, *fimF*, *fimG*, *fimH*, *fimI*, and *fimK*) were highly represented in all samples. The *fim* genes cluster is the key virulence factor responsible for the production of type 1 pili, where *fimA* is the major subunit component structuring the type 1 pili [30], *fimH* is the mannose-binding adhesion, *fimI* is essential for the construction of type 1 fimbriae despite that its product is of unknown function, *fimE* turns the expression from”on’’ to “off”, *fimB* turns the expression to either directions, while *fimC* encodes fimbrial chaperone, *fimD* encodes the fimbrial usher protein, *fimK* is unique for the *K*. *pneumonia* and is located downstream to the *fimH* playing a role in the regulation of the fimbrial expression, and *fimF*, *fimG* and *fimH* are the minor components [31]. It is known that type 3 fimbriae belong to the chaperone-usher class of fimbriae are encoded by five genes *mrk (A*, *B*, *C*, *D*, and *F*) [32]. Interestingly, we identified *mrk* (*A*, *B*, *C*, *D*, *F*, *H*, *I*, and *J*) in our samples which support the concept that they are highly virulent strains.

Other virulence factors that were identified including *fepB*, *fepC*, *fepD*, *and fepG* genes that are documented to be required for catecholate siderophores translocation in cytoplasm [33]. This iron uptake system that captures siderophores is one of the strategies used by bacteria to increase its pathogenesis as a determining factor in the outcome of infection [34]. Moreover, our results revealed another genes cluster coding for enterobactin synthesis (*entA*, *entB*, *entC*, *entE*, and *entF*). Enterobactin is the strongest siderophore known which acquires iron for bacterial systems raising the pathogenesis [34].

Another genes cluster was detected in our analysis which known as T6SS and contain 13 conserved core genes named *tss (A* to *M*). Here, we detected 10 from 13 genes *tss (B*, *C*, *D*, *F*, *G*, *H*, *J*, *K*, *L*, and *M*). They are encoding the proteins making up the basic secretion apparatus and producing a functioning system [35]. It has been proved earlier that bacteria contain T6SSs is easily manipulate host cells during pathogenesis and kill other competing bacteria, which, in some cases, increases horizontal gene transfer [36]. Other virulence determinants identified in this study were *wbbM*, *wbbN*, and *wbbO* that belonged to glycosyltransferase family and known to play a role in the biosynthesis of O-antigen which constitutes lipopoly saccharides contributing to biofilm formation [37]. Furthermore, *wzM* and *wzT* transmembrane transporters were identified that known to encode for a specific O12 ABC 2 export system: an ATP-binding cassette transporter for O-polysaccharide biosynthesis [38]. Hence, the presence of *wbb* and *wz* are confirming again that our isolates are highly pathogenic. Adding to these virulence factors, we detected *ybt* locus which was detected previously in 40% of *K*. *pneumoniae* genomes, particularly amongst those associated with invasive infections, promoting respiratory tract infections through evasion of Lcn2 [39].

Capsular or lipopolysaccharide typing is considered of importance since it provides extra- categorization that limits the characterization of *K*. *pneumoniae* in comparison with the sequence typing solely. Previous study disclosed that *K*. *pneumoniae* with the same sequence type but with different capsular types showed different characters [40]. Giving more credence to the fact that capsular typing might be used for a further differentiation of *K*. *pneumoniae* especially in clinical settings, which is a critical quest in epidemiological surveillance and infection prevention [40]. It has been recorded more than 130 capsular types up to now based on the available genomic data of *K*. *pneumonia* [41]. Notwithstanding, the capsular typing of the four samples included in this study according to the *wze* and *wzi* genes has revealed that the four strains are of K type (KL-24) and of O type (O1v1). Previous study has correlated KL-24 with clonal group 45 (CG-45) and O1v1 with ST 231 in clinical isolates of *K*. *pneumonia* [42]. Herein we announced a strong association between *K*. *pneumoniae* ST 627 and KL-24 and O1v1 capsular types.

At the same time, one plasmid was found in all four samples known as *pC17KP0052-1* and another plasmid with accession no. NZ_CP032191.1 was found only in K90. *IncFIB(K)* and *IncFII(K)* are two replicons found in all samples, while *ColRNAI* replicon was found only in K90 (S3 and S4 Tables). One of the most frequent replicons found in all samples, *IncFIB(K) (pKPN-IT)*, has been documented to be related to some virulence-associated genes including *fimH* that has a high adhesion ability which promote the bacterial pathogenicity [43, 44]. Also, it was reported that *IncFIB(K) (pKPN-IT)* has been conferred to arsenic, copper, silver, trimethoprim, streptomycin, chloramphenicol, and macrolide resistance [45]. Coincident with what was reported about plasmid *pKPN-CZ* that disclosed an elevated number of virulence encoding clusters relative to different plasmids formerly detected in *K*. *pneumoniae* strains [46]. The other most frequent replicon found in our study, *IncFII(K)*, has been recently proved to co-carry *bla*IMP–26 and tigecycline-resistance gene variant in a clinical *K*. *pneumoniae* isolate which displayed resistance to carbapenems and tigecycline [47]. There is a general agreement that a hallmark of *Inc*-family plasmids is often correlated with multi drug resistance (MDR) and virulence factors [48]. The two plasmids found in our analysis originated from *K*. *pneumoniae* (S3 Table).

The other mobile genetic elements that were detected in our study are the insertion sequences. Thirteen insertion sequences have been revealed with the analysis using ISfinder (Fig 1(C) and S5 Table); IS*1R*, IS*1X2*, IS*26*, IS*903B*, IS*Ec15*, IS*Ecl1*, IS*Kpn1*, IS*Kpn14*, IS*Kpn26*, IS*Kpn28*, IS*Kpn49*, IS*Kpn54*, and IS*Sen9*. The most frequent insertion sequence that was highly found in all of the four samples is IS*26* which is participated in the mobilization of wide range of antibiotic resistance genes, is played a critical role in the evolution of multidrug resistant (MDR) phenotypes in Enterobacteriales family and is known to be preferable location for translocable units [49]. The second most frequent insertion sequence is IS*Kpn26* that was documented to take part in increasing colistin resistance in *K*. *pneumoniae* along with IS*903B* [50]. Other insertion sequences that were identified in our study with biological roles including IS*1X2* which is known to cause resistance of zidovudine [51], IS*1R* that is known as a multiple drug resistance plasmid [52], and IS*Kpn1* which belongs to the IS*3* family and is known for its transposition activity between plasmids and chromosomes of *K*. *pneumoniae* and *E*. *coli* [53]. The results reported here confirm the high possibility of spreading the resistance elements from *K*. *pneumoniae* ST627 as a reservoir for mobile genetic elements carrying many genes for antibiotic resistance.

For the analysis of the phage region, PHASTER tool was used to identify the presence of the bacteriophages. The results of prophage regions for all samples were shown in S6 Table. Three samples out of four; K04, K96, and K90, obtained one intact bacteriophage and 4 incomplete ones and the fourth sample; K75, has two complete bacteriophages and 3 incomplete ones. The intact bacteriophage identified in K04, K69, and K 90 is Entero *P88*. In sample K75, two intact bacteriophages were identified namely Salmon *SEN5* followed by Klebsi phiKO2. Our results were consistent with what was reported before for clinical isolates of *K*. *pneumoniae* that identified Entero *P88*, Salmon *SEN5*, and Klebsi phiKO2 intact phages alongside with incomplete phages [22].

**Fig 2:**
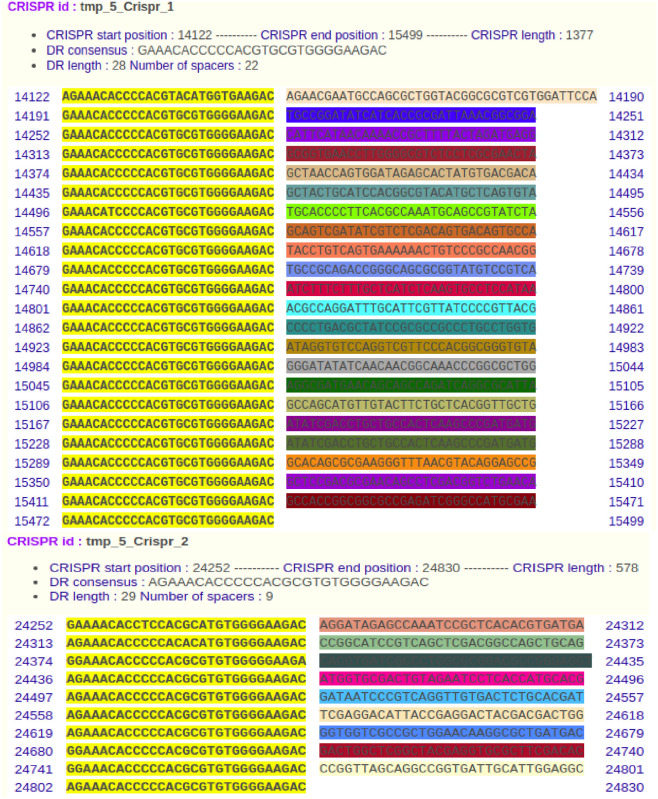
CRISPR arrays detected in the *K*. *pneumoniae* K04 as a representative of our samples. Two different characterization of CRISPR arrays detected including (CRISPR 1) and (CRISPR 2) in *K*. *pneumoniae* K04. CRISPR1 array is composed of 23 direct repeated sequences and 22 spacer sequences located at nucleotides 14122 to 15499. CRISPR2 array is composed of 10 direct repeated sequences and 9 spacer sequences located at nucleotides 24252 to 24830.

On the other hand, even the identification of Clustered Regularly Interspaced Palindromic Repeats (CRISPR) sequences has been detected in a very few *K*. *pneumoniae* isolates worldwide [22], our utilized high throughput biology technology has revealed that all the samples harbored CRISPR arrays including CRISPR1 and CRISPR2 using CRISPRFinder. In sample K04, as a representative, CRISPR1 and CRISPR2 were located from nucleotide 14122 to 15422 with 29 spacers and 24252 to 24830 with nine spacers, respectively (Fig 2). In sample K69, the two CRISPRs were located on two different contigs 14 and 44 from nucleotide 133988 to 134566 with nine spacers and 14122 to 15499 with 22 spacers, respectively. In sample K75, CRISPR1 and CRISPR2 were located from nucleotide 59694 to 61071 with 22 spacers and 69824 to 70402 with nine spacers, respectively. In sample K90, CRISPR1 and CRISPR2 were located from nucleotide 14122 to 15499 with 22 spacers and 24252 to 24830 with nine spacers, respectively. All CRISPRs in all samples were identified as CRISPR-associated *Cas3* helicases which are essential for the CRISPR-mediated adaptive immune systems in bacteria and archaea [54]. Adding to that, it is considered one of the bacterial defense mechanisms since it is implicated in bacterial protection against phage and various horizontal gene transfers through breaking down the plasmid DNA [55] that most likely unraveled the inverse relation in between the presence of CRISPR-associated *Cas* and the acquiring of antibiotics resistance [55]. Conversely, a striking observation of CRISPR-associated *Cas* in *K*. *pneumoniae* ST 152 and ST 607, highly resistant strains, was noted which contrariwise suggested the probable implication of CRISPR-associated *Cas3* in acquiring the resistance genes. This could be attributed to the presence of CRISPR-associated *Cas* between genes that encode proteins which are engaged in metabolism and antibiotics resistance [56]. Not only that, but also these two highly resistant strains were found to have many phages with the CRISPR-associated *cas* which in turn draw our attention for further investigations on the emergence and the transmission of antibiotic resistance [22].

**Fig 3:**
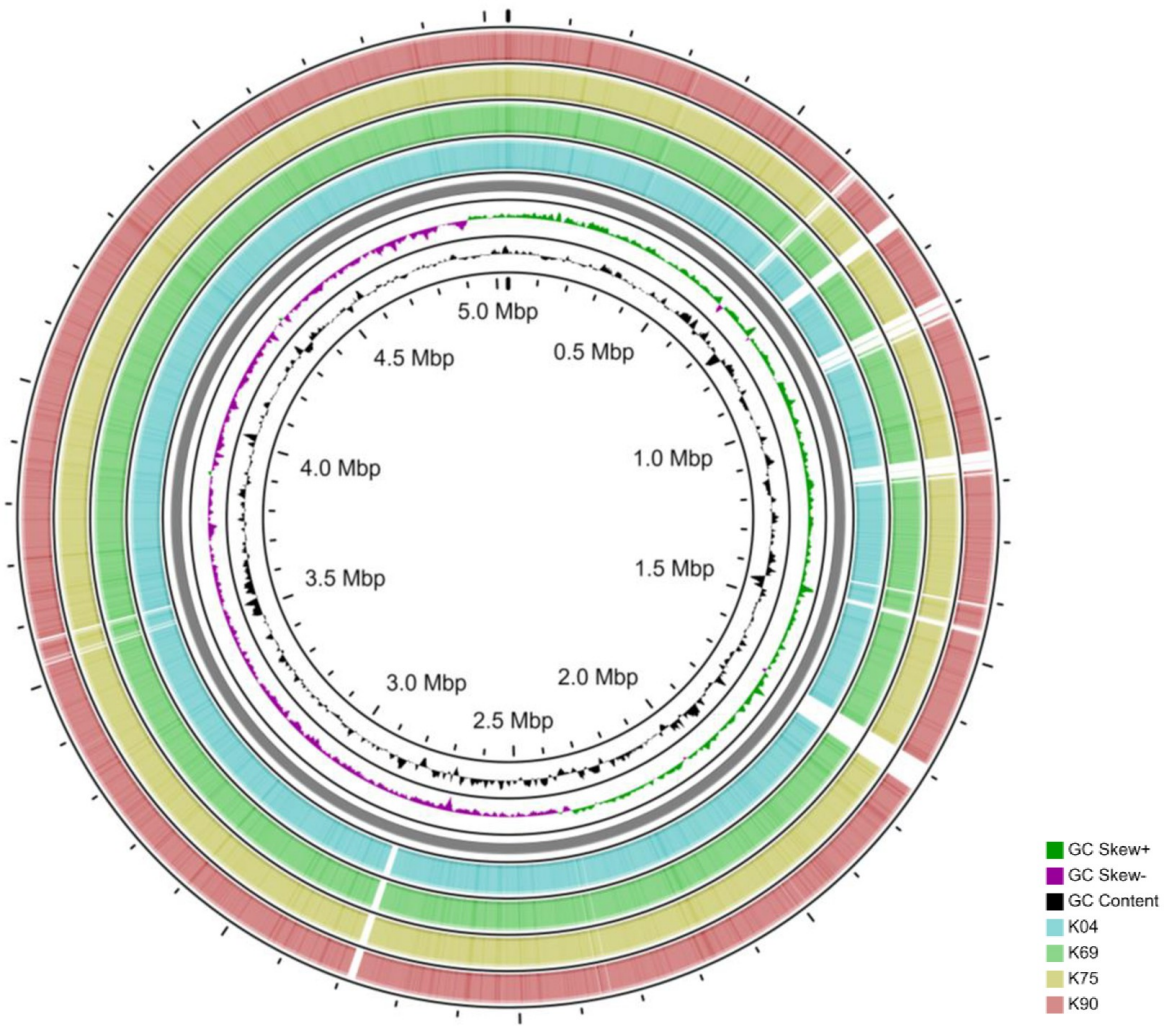
Circular genomic map comparing our isolates to the reference genome of *Klebsiella pneumoniae* strain C17KP0052 (Accession number: CP052388.1). The black histogram represents the CG content, and the green-purple histogram represents the CG skew.

Circular genomic map of the reference genome of *K*. *pneumoniae* strain C17KP0052 was shown in Fig 3. Phylogenetic tree for the reference genome and the core genome of our samples showed that K90 and K69 are belonged to the same genetic cluster, while K75 was the closest one to the reference genome used (S1 Fig). Variant calling analysis of the isolates revealed a total of 9783, 9832, 9810, and 9762 variants in K04, K69, K75, and K90, respectively. The 4 isolates were relatively similar in the SNPs across the core genome; there are 9667 common SNPs between the 4 isolates and 9, 28, 27, and 6 SNPs unique for K04, K69, K75 and K90, respectively (S7 Table).

Eventually, this study is the first comprehensive whole genome sequencing study about *K*. *pneumoniae* ST627 that is disseminating in Egypt as well as worldwide. We disclosed that the utmost prevalent antimicrobial resistance mechanism among our isolates was antibiotic efflux followed by antibiotic inactivation, antibiotic target replacement, and reduced permeability to antibiotics. The highly identified virulence factors were involved in pili formation and adhesion, stress tolerance, and capturing siderophores. Association between *K*. *pneumoniae* ST 627 and specific K and O serotypes was observed; KL-24 and O1v1; respectively. Our results shed the light on critical roles of the mobile genetic elements in ST 627 in the spreading of antibiotic resistance.

## Materials and methods

### Bacterial isolation and growth conditions

Four *K*. *pneumoniae* isolates sequence type 627 were used [14]; two of them were isolated from urine samples and two from blood specimens. Informed consent from each patient was obtained for study participation. Bacterial isolates were cultured on both MacConkey agar (Oxoid, Cambridge, UK) and then on Eosin Methylene Blue agar (Oxoid, Cambridge, UK). They were additionally identified using API 20 E system (BioMerieux). The strains were sub-cultured on Luria-Bertani broth (Oxoid, Cambridge, UK) at 37 °C for 24 h for DNA extraction.

### Bacterial DNA extraction

The bacterial cultures were pelleted by centrifugation for 10 min at 5000 x *g*. DNA was extracted using QIAamp^®^ DNA Mini kit (QIAGEN, Germany) according to the protocol for bacteria provided by the manufacturer and stored at -20°C till used for the preparation of the library.

### Library preparation and next generation sequencing

The preparation of the library was carried out utilizing the Nextera XT DNA Library preparation kit (Illumina, USA). The DNA was prepared, fragmented, and then tagged utilizing the transposome in the Nextera XT Kit. Unique adapters were compiled to each sample for labelling. PCR reaction of 12 cycles was done to amplify the DNA fragments to add primers and indices for dual-indexed sequencing of pooled libraries. Normalization of the samples followed by pooling and subjecting to 300-base paired-end reads sequencing with Illumina MiSeq platform were performed. All the preparation and sequencing were done following the manufacturer’s instructions.

### Genome assembly, annotation, and alignment

The quality of the generated read-pairs was checked using FastQC [57] and filtered using FastX toolkit (http://hannonlab.cshl.edu/fastx_toolkit/). After that, the filtered reads were merged using PEAR (https://doi.org/10.1093/bioinformatics/btt593) [58]. The merged reads were *de novo* assembled using SPAdes (https://doi.org/10.1089/cmb.2012.0021) [59] and aligned using BLAST (https://doi.org/10.1016/S0022-2836(05)80360-2) [60]. The assessment of the assembled files was carried out by QUAST (v5.0.2) [61] (https://doi.org/10.1093/bioinformatics/btt086) as shown in S8 Table.

### Genome analysis

The circular genome comparison was generated using CGView Server (http://cgview.ca/) (https://doi.org/10.1093/nar/gkn179) [62]. The detection of single-nucleotide polymorphisms (SNPs) and the identification of all variants (SNPs, insertions, and deletions) in our isolates were performed with Snippy (https://github.com/tseemann/snippy) [63].

Genome sequence of *Klebsiella pneumoniae* strain C17KP0052 (Accession number: CP052388.1) was used as a reference. After variant calling, the phylogeny tree was generated using FastTree [64] (http://www.microbesonline.org/fasttree/) (https://doi.org/10.1093/molbev/msp077).

### Identification of resistance determinants, virulence factors, mobile genetic elements, and capsular typing

The profiling of AMR genes and drug classes was performed by aligning the reads against the ResFinder database (https://cge.cbs.dtu.dk/services/ResFinder/) (https://doi.org/10.1093/jac/dks261) [65]. Plasmid carrying virulence determinants and insertion sequences were investigated by aligning the reads to the Virulence Factors Database (VFDB, http://www.mgc.ac.cn/VFs/) (https://doi.org/10.1093/nar/gki008) [66] and ISFinder database (https://github.com/thanhleviet/ISfinder-sequences) (https://doi.org/10.1093/nar/gkj014) [67], respectively. In addition, plasmids and replicons were characterized by aligning the contigs of all the samples versus the plasmid database (PLSDB, https://ccb-microbe.cs.uni-saarland.de/plsdb/) (https://doi.org/10.1093/nar/gky1050) [68] and the reads against PlasmidFinder database (https://cge.cbs.dtu.dk/services/PlasmidFinder/) [69], respectively. The identification and visualization of the phage regions and Clustered Regularly Interspaced Short Palindromic Repeats (CRISPR) were performed using PHASTER (https://phaster.ca/) (https://doi.org/10.1093/nar/gkw387) and CRISPRFinder (https://crispr.i2bc.paris-saclay.fr/Server/) (https://doi.org/10.1093/nar/gkm360), respectively [70, 71]. For capsular typing, Kaptive (https://kaptive-web.erc.monash.edu/) (https://jcm.asm.org/content/56/6/e00197-18.short) was used to determine K-type and O-type of each isolate [72].

### Downstream analysis and visualization

Alignments results were imported to R-studio (https://www.jstor.org/stable/41337225) [73] for further analysis. Reads with ratio < 97% identity or 1e−4 e-value were filtered out. The gene coverage was determined as the percentage of covered bases in each gene. Calculation of the gene copy number was performed through dividing gene reads number by the gene length. The mean coverage of each gene in all isolates was calculated for further validation of the computational methods. Cut-off coverage of 90% was chosen and the genes with sequencing reads covering > 90% of their length were only included in the downstream analyses. The heatmaps was generated using gplots package (https://www.rdocumentation.org/packages/gplots) [74].

### Compliance with ethical standards

This study was approved by Suez Canal University ethical board (No. 201612R3). All experiments were performed in accordance with relevant guidelines and regulations.

## Supporting information

S1 Table Profiling of antimicrobial resistance (AMR) genes, antibiotic class and the resistance mechanisms. (DOCX)

S2 Table Virulence factors profiling of the four samples belonged to ST 627. (DOCX)

S3 Table Plasmids identified in the four samples belonged to ST 627. (DOCX)

S4 Table Replicons identified in the plasmids of the four samples belonged to ST 627. (DOCX)

S5 Table Insertion sequences identified in the four samples belonged to ST 627. (DOCX)

S6 Table Prophage regions identified in the four samples belonged to ST 627. (DOCX)

S7 Table SNPs across the core genome of the four samples belonged to ST 627. (DOCX)

S8 Table The assessment of the assembled files was carried out by QUAST. (DOCX)

S1 Fig Phylogenetic analysis of the core genome for the four samples belonged to ST 627. (DOCX)
