# Supplementary material for: Whole genome sequencing of Klebsiella pneumoniae clinical isolates sequence type 627 isolated from Egyptian patients
Source: PLoS One. 2022 Mar 23;17(3):e0265884. doi: 10.1371/journal.pone.0265884 (PMC8942217; doi:10.1371/journal.pone.0265884)
Supplement: S1 Table — (DOCX) [file pone.0265884.s001.docx]

**S1 Table: Profiling of antimicrobial resistance (AMR) genes, antibiotic class and the resistance mechanisms**

| **Isolate** | **Gene** | **Drug Class** | **Resistance Mechanism** | **Reads Count** | **Coverage** | **Copy Number** |
| --- | --- | --- | --- | --- | --- | --- |
| K04 | *oqxA* | Quinolone | Antibiotic Efflux | 109 | 100 | 0.092687075 |
| K04 | *oqxB* | Quinolone | Antibiotic Efflux | 302 | 100 | 0.095781795 |
| K04 | *tet(D)* | Tetracycline | Antibiotic Efflux | 272 | 100 | 0.114767932 |
| K04 | *aph(3'')-Ib* | Aminoglycoside | Antibiotic Inactivation | 71 | 100 | 0.088308458 |
| K04 | *aph(6)-Id* | Aminoglycoside | Antibiotic Inactivation | 276 | 100 | 0.110180908 |
| K04 | *blaTEM-234* | Beta-lactam | Antibiotic Inactivation | 70 | 95.59 | 0.081300813 |
| K04 | *fosA* | Fosfomycin | Antibiotic Inactivation | 30 | 90.95 | 0.035714286 |
| K04 | *fosA6* | Fosfomycin | Antibiotic Inactivation | 45 | 97.69 | 0.103926097 |
| K04 | *sul2* | Sulfonamide | Antibiotic Target Replacement | 234 | 100 | 0.095588235 |
| K69 | *oqxA* | Quinolone | Antibiotic Efflux | 137 | 100 | 0.116496599 |
| K69 | *oqxB* | Quinolone | Antibiotic Efflux | 311 | 100 | 0.098636219 |
| K69 | *tet(D)* | Tetracycline | Antibiotic Efflux | 424 | 100 | 0.178902954 |
| K69 | *aph(3'')-Ib* | Aminoglycoside | Antibiotic Inactivation | 114 | 100 | 0.141791045 |
| K69 | *aph(6)-Id* | Aminoglycoside | Antibiotic Inactivation | 752 | 100 | 0.225017145 |
| K69 | *blaTEM-234* | Beta-lactam | Antibiotic Inactivation | 125 | 95.47 | 0.145180023 |
| K69 | *fosA6* | Fosfomycin | Antibiotic Inactivation | 54 | 97.69 | 0.124711316 |
| K69 | *sul2* | Sulfonamide | Antibiotic Target Replacement | 333 | 100 | 0.136029412 |
| K75 | *oqxA* | Quinolone | Antibiotic Efflux | 138 | 100 | 0.117346939 |
| K75 | *oqxB* | Quinolone | Antibiotic Efflux | 325 | 100 | 0.103076435 |
| K75 | *tet(D)* | Tetracycline | Antibiotic Efflux | 330 | 100 | 0.139240506 |
| K75 | *aph(3'')-Ib* | Aminoglycoside | Antibiotic Inactivation | 67 | 100 | 0.083333333 |
| K75 | *aph(6)-Id* | Aminoglycoside | Antibiotic Inactivation | 345 | 100 | 0.137726135 |
| K75 | *blaTEM-234* | Beta-lactam | Antibiotic Inactivation | 77 | 95.12 | 0.089430894 |
| K75 | *fosA6* | Fosfomycin | Antibiotic Inactivation | 56 | 97.69 | 0.129330254 |
| K75 | *sul2* | Sulfonamide | Antibiotic Target Replacement | 140 | 100 | 0.085784314 |
| K90 | *oqxA* | Quinolone | Antibiotic Efflux | 153 | 100 | 0.130102041 |
| K90 | *oqxB* | Quinolone | Antibiotic Efflux | 295 | 100 | 0.093561687 |
| K90 | *tet(D)* | Tetracycline | Antibiotic Efflux | 296 | 100 | 0.124894515 |
| K90 | *aph(3'')-Ib* | Aminoglycoside | Antibiotic Inactivation | 86 | 100 | 0.106965174 |
| K90 | *aph(6)-Id* | Aminoglycoside | Antibiotic Inactivation | 243 | 100 | 0.097007104 |
| K90 | *blaTEM-234* | Beta-lactam | Antibiotic Inactivation | 65 | 95.70 | 0.075493612 |
| K90 | *fosA6* | Fosfomycin | Antibiotic Inactivation | 44 | 97.69 | 0.101616628 |
| K90 | *sul2* | Sulfonamide | Antibiotic Target Replacement | 114 | 100 | 0.069852941 |
