# Supplementary material for: Whole genome sequencing of Klebsiella pneumoniae clinical isolates sequence type 627 isolated from Egyptian patients
Source: PLoS One. 2022 Mar 23;17(3):e0265884. doi: 10.1371/journal.pone.0265884 (PMC8942217; doi:10.1371/journal.pone.0265884)
Supplement: S2 Table — (DOCX) [file pone.0265884.s002.docx]

**S2 Table: Virulence factors profiling of the four isolates belonged to ST 627**

| **Isolate** | **VF** | **Reads Count** | **Coverage** | **Copy Number** | **Isolate** | **VF** | **Reads Count** | **Coverage** | **Copy Number** |
| --- | --- | --- | --- | --- | --- | --- | --- | --- | --- |
| K04 | *acrA* | 164 | 100 | 0.137353434 | K75 | *acrA* | 154 | 100 | 0.128978224 |
| K04 | *acrB* | 395 | 100 | 0.125516365 | K75 | *acrB* | 454 | 100 | 0.144264379 |
| K04 | *clpV/tssH* | 265 | 100 | 0.099811676 | K75 | *clpV/tssH* | 228 | 100 | 0.085875706 |
| K04 | *dotU/tssL* | 101 | 100 | 0.146376812 | K75 | *dotU/tssL* | 108 | 100 | 0.156521739 |
| K04 | *entA* | 87 | 100 | 0.110687023 | K75 | *entA* | 81 | 100 | 0.103053435 |
| K04 | *entB* | 83 | 100 | 0.09741784 | K75 | *entB* | 68 | 100 | 0.079812207 |
| K04 | *entC* | 97 | 100 | 0.081649832 | K75 | *entC* | 88 | 100 | 0.074074074 |
| K04 | *entE* | 129 | 97.70 | 0.080223881 | K75 | *entE* | 102 | 97.39 | 0.063432836 |
| K04 | *entF* | 327 | 100 | 0.08423493 | K75 | *entF* | 314 | 100 | 0.080886141 |
| K04 | *fepB* | 103 | 100 | 0.107291667 | K75 | *fepB* | 105 | 100 | 0.109375 |
| K04 | *fepC* | 88 | 100 | 0.110691824 | K75 | *fepC* | 98 | 100 | 0.12327044 |
| K04 | *fepD* | 87 | 100 | 0.086309524 | K75 | *fepD* | 99 | 100 | 0.098214286 |
| K04 | *fepG* | 81 | 100 | 0.081570997 | K75 | *fepG* | 108 | 100 | 0.108761329 |
| K04 | *fes* | 149 | 100 | 0.123242349 | K75 | *fes* | 142 | 100 | 0.11745244 |
| K04 | *fimA* | 78 | 100 | 0.142076503 | K75 | *fimA* | 65 | 100 | 0.118397086 |
| K04 | *fimB* | 83 | 100 | 0.136963696 | K75 | *fimB* | 103 | 100 | 0.169966997 |
| K04 | *fimC* | 87 | 100 | 0.119834711 | K75 | *fimC* | 87 | 100 | 0.119834711 |
| K04 | *fimD* | 376 | 100 | 0.142101285 | K75 | *fimD* | 380 | 100 | 0.143613001 |
| K04 | *fimE* | 85 | 100 | 0.139573071 | K75 | *fimE* | 63 | 100 | 0.103448276 |
| K04 | *fimF* | 43 | 100 | 0.080979284 | K75 | *fimF* | 42 | 100 | 0.079096045 |
| K04 | *fimG* | 61 | 100 | 0.121756487 | K75 | *fimG* | 80 | 100 | 0.159680639 |
| K04 | *fimH* | 136 | 100 | 0.149614961 | K75 | *fimH* | 135 | 100 | 0.148514851 |
| K04 | *fimI* | 96 | 100 | 0.150943396 | K75 | *fimI* | 71 | 100 | 0.11163522 |
| K04 | *fimK* | 204 | 100 | 0.164251208 | K75 | *fimK* | 163 | 100 | 0.131239936 |
| K04 | *fyuA* | 242 | 100 | 0.119683482 | K75 | *fyuA* | 232 | 100 | 0.114737883 |
| K04 | *galF* | 125 | 100 | 0.1393534 | K75 | *galF* | 124 | 100 | 0.138238573 |
| K04 | *glf* | 95 | 100 | 0.082251082 | K75 | *glf* | 111 | 100 | 0.096103896 |
| K04 | *hcp/tssD* | 60 | 100 | 0.12195122 | K75 | *hcp/tssD* | 82 | 100 | 0.166666667 |
| K04 | *iroE* | 89 | 100 | 0.09508547 | K75 | *icmF/tssM* | 249 | 90.02 | 0.07267951 |
| K04 | *irp1* | 982 | 100 | 0.103455542 | K75 | *iroE* | 97 | 100 | 0.103632479 |
| K04 | *irp2* | 617 | 100 | 0.101015062 | K75 | *irp1* | 935 | 100 | 0.098504003 |
| K04 | *manB* | 139 | 100 | 0.10138585 | K75 | *irp2* | 631 | 100 | 0.103307138 |
| K04 | *manC* | 136 | 100 | 0.096045198 | K75 | *manB* | 169 | 100 | 0.123267688 |
| K04 | *mrkA* | 71 | 100 | 0.116584565 | K75 | *manC* | 141 | 100 | 0.099576271 |
| K04 | *mrkB* | 61 | 100 | 0.086894587 | K75 | *mrkA* | 86 | 100 | 0.141215107 |
| K04 | *mrkC* | 321 | 100 | 0.12907117 | K75 | *mrkB* | 61 | 100 | 0.086894587 |
| K04 | *mrkD* | 116 | 100 | 0.116465863 | K75 | *mrkC* | 341 | 100 | 0.137112988 |
| K04 | *mrkF* | 54 | 100 | 0.08490566 | K75 | *mrkD* | 124 | 100 | 0.124497992 |
| K04 | *mrkH* | 67 | 100 | 0.094233474 | K75 | *mrkF* | 57 | 100 | 0.089622642 |
| K04 | *mrkI* | 62 | 100 | 0.105982906 | K75 | *mrkH* | 66 | 100 | 0.092827004 |
| K04 | *mrkJ* | 73 | 100 | 0.10181311 | K75 | *mrkI* | 60 | 100 | 0.102564103 |
| K04 | *rcsA* | 51 | 100 | 0.081730769 | K75 | *mrkJ* | 70 | 100 | 0.09762901 |
| K04 | *rcsB* | 74 | 100 | 0.113671275 | K75 | *rcsA* | 52 | 100 | 0.083333333 |
| K04 | *sciN/tssJ* | 44 | 100 | 0.081031308 | K75 | *rcsB* | 72 | 100 | 0.110599078 |
| K04 | *tssF* | 158 | 100 | 0.09002849 | K75 | *sciN/tssJ* | 44 | 100 | 0.081031308 |
| K04 | *tssG* | 78 | 100 | 0.071823204 | K75 | *tssF* | 147 | 100 | 0.083760684 |
| K04 | *ugd* | 76 | 95.37 | 0.06512425 | K75 | *tssG* | 86 | 100 | 0.079189687 |
| K04 | *vasE/tssK* | 130 | 100 | 0.09672619 | K75 | *ugd* | 76 | 94.00 | 0.06512425 |
| K04 | *vipA/tssB* | 22 | 100 | 0.044715447 | K75 | *vasE/tssK* | 107 | 100 | 0.079613095 |
| K04 | *vipB/tssC* | 164 | 100 | 0.106148867 | K75 | *vipA/tssB* | 27 | 100 | 0.054878049 |
| K04 | *wbbM* | 127 | 100 | 0.066983122 | K75 | *vipB/tssC* | 185 | 100 | 0.1197411 |
| K04 | *wbbN* | 81 | 100 | 0.090604027 | K75 | *wbbM* | 128 | 100 | 0.067510549 |
| K04 | *wbbO* | 47 | 92.48 | 0.041556145 | K75 | *wbbN* | 80 | 100 | 0.089485459 |
| K04 | *wzm* | 36 | 100 | 0.046153846 | K75 | *wbbO* | 60 | 92.48 | 0.053050398 |
| K04 | *wzt* | 66 | 100 | 0.089068826 | K75 | *wzm* | 27 | 100 | 0.034615385 |
| K04 | *ybdA* | 139 | 100 | 0.111916264 | K75 | *wzt* | 56 | 100 | 0.075573549 |
| K04 | *ybtA* | 122 | 100 | 0.127083333 | K75 | *ybdA* | 132 | 100 | 0.106280193 |
| K04 | *ybtE* | 168 | 100 | 0.106463878 | K75 | *ybtA* | 99 | 100 | 0.103125 |
| K04 | *ybtP* | 169 | 100 | 0.093732668 | K75 | *ybtE* | 167 | 100 | 0.105830165 |
| K04 | *ybtQ* | 161 | 99.26 | 0.08863688 | K75 | *ybtP* | 178 | 100 | 0.098724348 |
| K04 | *ybtS* | 82 | 95.17 | 0.062835249 | K75 | *ybtQ* | 188 | 99.26 | 0.10350145 |
| K04 | *ybtT* | 87 | 100 | 0.108208955 | K75 | *ybtS* | 75 | 93.26 | 0.057471264 |
| K04 | *ybtU* | 126 | 97.42 | 0.111484274 | K75 | *ybtT* | 78 | 100 | 0.097014925 |
| K04 | *ybtX* | 107 | 95.62 | 0.07986966 | K75 | *ybtU* | 122 | 97.42 | 0.107945091 |
| K69 | *acrA* | 163 | 100 | 0.136515913 | K75 | *ybtX* | 117 | 95.62 | 0.087334114 |
| K69 | *acrB* | 419 | 100 | 0.133142676 | K90 | *acrA* | 174 | 100 | 0.145728643 |
| K69 | *clpV/tssH* | 271 | 100 | 0.102071563 | K90 | *acrB* | 407 | 100 | 0.12932952 |
| K69 | *dotU/tssL* | 101 | 100 | 0.146376812 | K90 | *clpV/tssH* | 230 | 100 | 0.086629002 |
| K69 | *entA* | 97 | 98.85 | 0.123409669 | K90 | *dotU/tssL* | 101 | 100 | 0.146376812 |
| K69 | *entB* | 86 | 100 | 0.100938967 | K90 | *entA* | 79 | 100 | 0.100508906 |
| K69 | *entC* | 104 | 100 | 0.087542088 | K90 | *entB* | 86 | 100 | 0.100938967 |
| K69 | *entE* | 118 | 96.83 | 0.073383085 | K90 | *entC* | 108 | 100 | 0.090909091 |
| K69 | *entF* | 372 | 100 | 0.095826893 | K90 | *entE* | 112 | 99.69 | 0.069651741 |
| K69 | *fepB* | 107 | 100 | 0.111458333 | K90 | *entF* | 326 | 100 | 0.083977331 |
| K69 | *fepC* | 87 | 100 | 0.109433962 | K90 | *fepB* | 133 | 100 | 0.138541667 |
| K69 | *fepD* | 108 | 100 | 0.107142857 | K90 | *fepC* | 101 | 100 | 0.127044025 |
| K69 | *fepG* | 96 | 100 | 0.096676737 | K90 | *fepD* | 87 | 100 | 0.086309524 |
| K69 | *fes* | 152 | 100 | 0.125723739 | K90 | *fepG* | 101 | 100 | 0.101711984 |
| K69 | *fimA* | 81 | 100 | 0.147540984 | K90 | *fes* | 142 | 100 | 0.11745244 |
| K69 | *fimB* | 81 | 100 | 0.133663366 | K90 | *fimA* | 75 | 100 | 0.136612022 |
| K69 | *fimC* | 91 | 100 | 0.125344353 | K90 | *fimB* | 89 | 100 | 0.146864686 |
| K69 | *fimD* | 415 | 100 | 0.156840514 | K90 | *fimC* | 95 | 100 | 0.130853994 |
| K69 | *fimE* | 88 | 100 | 0.144499179 | K90 | *fimD* | 429 | 100 | 0.162131519 |
| K69 | *fimF* | 57 | 100 | 0.107344633 | K90 | *fimE* | 76 | 100 | 0.124794745 |
| K69 | *fimG* | 78 | 100 | 0.155688623 | K90 | *fimF* | 49 | 100 | 0.092278719 |
| K69 | *fimH* | 144 | 100 | 0.158415842 | K90 | *fimG* | 75 | 100 | 0.149700599 |
| K69 | *fimI* | 88 | 100 | 0.13836478 | K90 | *fimH* | 128 | 100 | 0.140814081 |
| K69 | *fimK* | 200 | 100 | 0.161030596 | K90 | *fimI* | 74 | 100 | 0.116352201 |
| K69 | *fyuA* | 271 | 100 | 0.134025717 | K90 | *fimK* | 184 | 100 | 0.148148148 |
| K69 | *galF* | 104 | 100 | 0.115942029 | K90 | *fyuA* | 281 | 100 | 0.138971316 |
| K69 | *glf* | 99 | 100 | 0.085714286 | K90 | *galF* | 117 | 100 | 0.130434783 |
| K69 | *hcp/tssD* | 85 | 100 | 0.172764228 | K90 | *glf* | 72 | 100 | 0.062337662 |
| K69 | *icmF/tssM* | 278 | 90.02 | 0.081144191 | K90 | *hcp/tssD* | 76 | 100 | 0.154471545 |
| K69 | *iroE* | 105 | 100 | 0.112179487 | K90 | *iroE* | 110 | 100 | 0.117521368 |
| K69 | *irp1* | 1072 | 100 | 0.11293721 | K90 | *irp1* | 946 | 100 | 0.099662874 |
| K69 | *irp2* | 694 | 100 | 0.11362148 | K90 | *irp2* | 618 | 100 | 0.101178782 |
| K69 | *manB* | 151 | 100 | 0.110138585 | K90 | *manB* | 145 | 100 | 0.105762217 |
| K69 | *manC* | 120 | 100 | 0.084745763 | K90 | *manC* | 135 | 100 | 0.095338983 |
| K69 | *mrkA* | 77 | 100 | 0.126436782 | K90 | *mrkA* | 80 | 100 | 0.13136289 |
| K69 | *mrkB* | 68 | 100 | 0.096866097 | K90 | *mrkB* | 63 | 100 | 0.08974359 |
| K69 | *mrkC* | 354 | 100 | 0.142340169 | K90 | *mrkC* | 334 | 100 | 0.134298351 |
| K69 | *mrkD* | 129 | 100 | 0.129518072 | K90 | *mrkD* | 108 | 100 | 0.108433735 |
| K69 | *mrkF* | 63 | 100 | 0.099056604 | K90 | *mrkF* | 64 | 100 | 0.100628931 |
| K69 | *mrkH* | 76 | 100 | 0.106891702 | K90 | *mrkH* | 67 | 100 | 0.094233474 |
| K69 | *mrkI* | 67 | 100 | 0.114529915 | K90 | *mrkI* | 66 | 100 | 0.112820513 |
| K69 | *mrkJ* | 71 | 100 | 0.09902371 | K90 | *mrkJ* | 71 | 100 | 0.09902371 |
| K69 | *rcsA* | 44 | 100 | 0.070512821 | K90 | *rcsA* | 41 | 100 | 0.065705128 |
| K69 | *rcsB* | 77 | 100 | 0.11827957 | K90 | *rcsB* | 93 | 100 | 0.142857143 |
| K69 | *sciN/tssJ* | 37 | 100 | 0.068139963 | K90 | *sciN/tssJ* | 40 | 100 | 0.073664825 |
| K69 | *tssF* | 169 | 100 | 0.096296296 | K90 | *tssF* | 132 | 100 | 0.075213675 |
| K69 | *tssG* | 78 | 100 | 0.071823204 | K90 | *tssG* | 90 | 100 | 0.082872928 |
| K69 | *ugd* | 79 | 91.35 | 0.067694944 | K90 | *ugd* | 91 | 93.32 | 0.077977721 |
| K69 | *vasE/tssK* | 121 | 100 | 0.090029762 | K90 | *vasE/tssK* | 111 | 100 | 0.082589286 |
| K69 | *vipA/tssB* | 27 | 100 | 0.054878049 | K90 | *vipA/tssB* | 18 | 100 | 0.036585366 |
| K69 | *vipB/tssC* | 172 | 100 | 0.111326861 | K90 | *vipB/tssC* | 154 | 100 | 0.099676375 |
| K69 | *wbbM* | 121 | 100 | 0.063818565 | K90 | *wbbM* | 120 | 100 | 0.063291139 |
| K69 | *wbbN* | 75 | 100 | 0.083892617 | K90 | *wbbN* | 70 | 100 | 0.078299776 |
| K69 | *wbbO* | 60 | 92.48 | 0.053050398 | K90 | *wbbO* | 54 | 92.48 | 0.047745358 |
| K69 | *wzm* | 36 | 100 | 0.046153846 | K90 | *wzm* | 28 | 100 | 0.035897436 |
| K69 | *wzt* | 70 | 100 | 0.094466937 | K90 | *wzt* | 67 | 100 | 0.090418354 |
| K69 | *ybdA* | 180 | 100 | 0.144927536 | K90 | *ybdA* | 141 | 100 | 0.11352657 |
| K69 | *ybtA* | 135 | 100 | 0.140625 | K90 | *ybtA* | 118 | 100 | 0.122916667 |
| K69 | *ybtE* | 204 | 100 | 0.129277567 | K90 | *ybtE* | 181 | 100 | 0.114702155 |
| K69 | *ybtP* | 180 | 100 | 0.099833611 | K90 | *ybtP* | 173 | 100 | 0.095951192 |
| K69 | *ybtQ* | 180 | 99.26 | 0.099097133 | K90 | *ybtQ* | 170 | 99.26 | 0.093591737 |
| K69 | *ybtS* | 79 | 91.34 | 0.060536398 | K90 | *ybtS* | 77 | 97.93 | 0.059003831 |
| K69 | *ybtT* | 95 | 100 | 0.118159204 | K90 | *ybtT* | 83 | 100 | 0.103233831 |
| K69 | *ybtU* | 123 | 97.42 | 0.108829887 | K90 | *ybtU* | 122 | 97.42 | 0.107945091 |
| K69 | *ybtX* | 105 | 95.62 | 0.078376769 | K90 | *ybtX* | 113 | 95.62 | 0.084348332 |
