# Supplementary material for: Whole genome sequencing of Klebsiella pneumoniae clinical isolates sequence type 627 isolated from Egyptian patients
Source: PLoS One. 2022 Mar 23;17(3):e0265884. doi: 10.1371/journal.pone.0265884 (PMC8942217; doi:10.1371/journal.pone.0265884)
Supplement: S3 Table — (DOCX) [file pone.0265884.s003.docx]

**S3 Table: Plasmids identified in the four isolates belonged to ST 627**

| **Isolate** | **Plasmid** | **Coverage** | **Contigs Count** | **Copy Number** | **Source Organism** |
| --- | --- | --- | --- | --- | --- |
| K04 | CP052389.1 (pC17KP0052-1) | 99.80 | 8 | 3.29E-05 | *Klebsiella pneumoniae* |
| K69 | CP052389.1 (pC17KP0052-1) | 99.80 | 8 | 3.29E-05 | *Klebsiella pneumoniae* |
| K75 | CP052389.1 (pC17KP0052-1) | 99.80 | 8 | 3.29E-05 | *Klebsiella pneumoniae* |
| K90 | CP052389.1 (pC17KP0052-1) | 99.80 | 8 | 3.29E-05 | *Klebsiella pneumoniae* |
| K90 | NZ_CP032191.1 (unnamed6) | 91.11 | 6 | 7.67E-05 | *Klebsiella pneumoniae* |
