# Supplementary material for: Whole genome sequencing of Klebsiella pneumoniae clinical isolates sequence type 627 isolated from Egyptian patients
Source: PLoS One. 2022 Mar 23;17(3):e0265884. doi: 10.1371/journal.pone.0265884 (PMC8942217; doi:10.1371/journal.pone.0265884)
Supplement: S4 Table — (DOCX) [file pone.0265884.s004.docx]

**S4 Table: Replicons identified in the plasmids of the four isolates belonged to ST 627**

| **Isolate** | **Replicon** | **Accession** | **Coverage** | **Reads Count** | **Copy Number** |
| --- | --- | --- | --- | --- | --- |
| K04 | IncFIB(K) | JN233704 | 100 | 87 | 0.155357143 |
| K04 | IncFII(K) | CP000648 | 100 | 6 | 0.040540541 |
| K69 | IncFIB(K) | JN233704 | 100 | 139 | 0.248214286 |
| K69 | IncFII(K) | CP000648 | 100 | 5 | 0.033783784 |
| K75 | IncFIB(K) | JN233704 | 100 | 89 | 0.158928571 |
| K75 | IncFII(K) | CP000648 | 100 | 2 | 0.013513514 |
| K90 | IncFIB(K) | JN233704 | 100 | 86 | 0.153571429 |
| K90 | ColRNAI | DQ298019 | 100 | 1 | 0.007692308 |
| K90 | IncFII(K) | CP000648 | 100 | 4 | 0.027027027 |
