# Supplementary material for: Whole genome sequencing of Klebsiella pneumoniae clinical isolates sequence type 627 isolated from Egyptian patients
Source: PLoS One. 2022 Mar 23;17(3):e0265884. doi: 10.1371/journal.pone.0265884 (PMC8942217; doi:10.1371/journal.pone.0265884)
Supplement: S5 Table — (DOCX) [file pone.0265884.s005.docx]

**S5 Table: Insertion sequences identified in the four isolates belonged to ST 627**

| **Isolate** | **IS** | **Group** | **Coverage** | **Reads Count** | **Copy Number** | **Isolate** | **IS** | **Group** | **Coverage** | **Reads Count** | **Copy Number** |
| --- | --- | --- | --- | --- | --- | --- | --- | --- | --- | --- | --- |
| K04 | ISKpn49 | IS66 | 91.55 | 221 | 0.090648072 | K75 | ISKpn1 | IS3 | 100 | 353 | 0.244291 |
| K04 | IS903B | IS5 | 96.59 | 296 | 0.280037843 | K75 | ISKpn49 | IS66 | 92.00 | 196 | 0.080394 |
| K04 | ISKpn1 | IS3 | 100 | 336 | 0.232525952 | K75 | IS26 | IS6 | 100 | 417 | 0.508537 |
| K04 | IS26 | IS6 | 100 | 415 | 0.506097561 | K75 | ISKpn54 | IS3 | 100 | 319 | 0.263419 |
| K04 | IS1X2 | IS1 | 100 | 167 | 0.217447917 | K75 | ISKpn26 | IS5 | 100 | 547 | 0.457358 |
| K04 | ISKpn54 | IS3 | 100 | 292 | 0.241123039 | K75 | ISKpn28 | IS481 | 100 | 115 | 0.104927 |
| K04 | ISEc15 | IS3 | 97.32 | 142 | 0.115447154 | K75 | ISEcl1 | IS3 | 94.69 | 129 | 0.096557 |
| K04 | ISKpn26 | IS5 | 100 | 469 | 0.392140468 | K75 | ISEc15 | IS3 | 100 | 157 | 0.127642 |
| K04 | ISEcl1 | IS3 | 98.35 | 153 | 0.114520958 | K75 | ISKpn14 | IS1 | 100 | 96 | 0.125 |
| K04 | ISKpn28 | IS481 | 100 | 139 | 0.126824818 | K75 | IS1X2 | IS1 | 100 | 195 | 0.253906 |
| K04 | ISKpn14 | IS1 | 100 | 137 | 0.178385417 | K90 | ISKpn1 | IS3 | 100 | 307 | 0.212457 |
| K04 | ISSen9 | IS1 | 97.27 | 32 | 0.041666667 | K90 | ISKpn26 | IS5 | 100 | 493 | 0.412207 |
| K69 | ISKpn14 | IS1 | 100 | 152 | 0.197916667 | K90 | IS26 | IS6 | 100 | 395 | 0.481707 |
| K69 | ISKpn26 | IS5 | 100 | 515 | 0.430602007 | K90 | ISKpn54 | IS3 | 100 | 204 | 0.168456 |
| K69 | ISKpn49 | IS66 | 90.94 | 295 | 0.12100082 | K90 | IS1X2 | IS1 | 100 | 170 | 0.221354 |
| K69 | IS26 | IS6 | 100 | 630 | 0.768292683 | K90 | ISKpn14 | IS1 | 100 | 109 | 0.141927 |
| K69 | ISKpn28 | IS481 | 100 | 182 | 0.166058394 | K90 | ISEcl1 | IS3 | 92.22 | 144 | 0.107784 |
| K69 | ISKpn1 | IS3 | 100 | 341 | 0.235986159 | K90 | ISKpn49 | IS66 | 90.98 | 190 | 0.077933 |
| K69 | IS903B | IS5 | 97.82 | 374 | 0.353831599 | K90 | ISEc15 | IS3 | 98.70 | 178 | 0.144715 |
| K69 | ISEc15 | IS3 | 100 | 224 | 0.182113821 | K90 | ISKpn28 | IS481 | 100 | 152 | 0.138686 |
| K69 | ISKpn54 | IS3 | 100 | 301 | 0.248554913 |  |  |  |  |  |  |
| K69 | ISEcl1 | IS3 | 99.10 | 188 | 0.140718563 |  |  |  |  |  |  |
| K69 | IS1X2 | IS1 | 100 | 220 | 0.286458333 |  |  |  |  |  |  |
| K69 | IS1R | IS1 | 92.58 | 51 | 0.06640625 |  |  |  |  |  |  |
| K69 | ISSen9 | IS1 | 98.83 | 60 | 0.078125 |  |  |  |  |  |  |
