# Supplementary material for: Whole genome sequencing of Klebsiella pneumoniae clinical isolates sequence type 627 isolated from Egyptian patients
Source: PLoS One. 2022 Mar 23;17(3):e0265884. doi: 10.1371/journal.pone.0265884 (PMC8942217; doi:10.1371/journal.pone.0265884)
Supplement: S6 Table — (DOCX) [file pone.0265884.s006.docx]

**S6 Table: prophage regions identified in the four isolates belonged to ST 627**

|  | **Region** | **Region Length** | **Completeness** | **Score** | **# Total Proteins** | **Region Position** | **Most Common Phage** | **GC %** |
| --- | --- | --- | --- | --- | --- | --- | --- | --- |
| **K04** | NODE_33_length_49830_cov_16.646866 | | | | | | | |
|  | 1 | 8.9Kb | incomplete | 40 | 12 | 40544-49542 | PHAGE_Entero_P1_NC_005856(2) | 60.23% |
|  | NODE_38_length_34645_cov_14.239434 | | | | | | | |
|  | 2 | 25.4Kb | intact | 150 | 29 | 1706-27161 | PHAGE_Entero_P88_NC_026014(18) | 53.15% |
|  | NODE_50_length_22089_cov_13.983855 | | | | | | | |
|  | 3 | 11.7Kb | incomplete | 30 | 14 | 464-12187 | PHAGE_Phage_Gifsy_2_NC_010393(5) | 53.80% |
|  | NODE_52_length_20824_cov_14.277759 | | | | | | | |
|  | 4 | 16.2Kb | incomplete | 30 | 12 | 1461-17737 | PHAGE_Salmon_SEN5_NC_028701(5) | 53.44% |
|  | NODE_54_length_20078_cov_14.513090 | | | | | | | |
|  | 5 | 18.7kb | incomplete | 30 | 32 | 1240-19959 | PHAGE_Escher_HK639_NC_016158(9) | 52.08% |
| **K69** | NODE_30_length_51812_cov_15.811607 | | | | | | | |
|  | 1 | 16.2Kb | incomplete | 30 | 13 | 34074-50362 | PHAGE_Salmon_SEN5_NC_028701(5) | 53.47% |
|  | NODE_31_length_49883_cov_21.466835 | | | | | | | |
|  | 2 | 8.9Kb | incomplete | 40 | 12 | 40597-49595 | PHAGE_Entero_P1_NC_005856(2) | 60.23% |
|  | NODE_38_length_34940_cov_15.302104 | | | | | | | |
|  | 3 | 25.4Kb | intact | 150 | 27 | 7485-32940 | PHAGE_Entero_P88_NC_026014(17) | 53.15% |
|  | NODE_46_length_22089_cov_15.302165 | | | | | | | |
|  | 4 | 20.7Kb | incomplete | 30 | 14 | 872-21626 | PHAGE_Phage_Gifsy_2_NC_010393(4) | 55.23% |
|  | NODE_50_length_20078_cov_15.278120 | | | | | | | |
|  | 5 | 18.7Kb | incomplete | 30 | 32 | 1240-19959 | PHAGE_Escher_HK639_NC_016158(9) | 52.08% |
| **K75** | NODE_21_length_86746_cov_14.330579 | | | | | | | |
|  | 1 | 37Kb | intact | 150 | 43 | 42268-79359 | PHAGE_Salmon_SEN5_NC_028701(20) | 50.79% |
|  | NODE_27_length_72136_cov_15.373791 | | | | | | | |
|  | 2 | 25.7Kb | incomplete | 50 | 15 | 9903-35626 | PHAGE_Phage_Gifsy_2_NC_010393(4) | 52.36% |
|  | 3 | 8.9Kb | incomplete | 40 | 13 | 62847-71845 | PHAGE_Entero_P1_NC_005856(2) | 60.23% |
|  | NODE_34_length_55052_cov_56.385962 | | | | | | | |
|  | 4 | 54.6Kb | intact | 110 | 55 | 430-55052 | PHAGE_Klebsi_phiKO2_NC_005857(46) | 52.52% |
|  | NODE_53_length_20078_cov_14.347550 | | | | | | | |
|  | 5 | 18.7Kb | incomplete | 30 | 34 | 120-18839 | PHAGE_Escher_HK639_NC_016158(9) | 52.08% |
| **K90** | NODE_30_length_51812_cov_14.821411 | | | | | | | |
|  | 1 | 16.2Kb | incomplete | 30 | 13 | 34074-50362 | PHAGE_Salmon_SEN5_NC_028701(5) | 53.47% |
|  | NODE_31_length_49883_cov_15.739585 | | | | | | | |
|  | 2 | 8.9Kb | incomplete | 40 | 12 | 40597-49595 | PHAGE_Entero_P1_NC_005856(2) | 60.23% |
|  | NODE_37_length_34951_cov_14.323329 | | | | | | | |
|  | 3 | 25.4Kb | intact | 150 | 29 | 2012-27467 | PHAGE_Entero_P88_NC_026014(18) | 53.15% |
|  | NODE_43_length_22089_cov_14.216118 | | | | | | | |
|  | 4 | 20.7Kb | incomplete | 30 | 14 | 872-21626 | PHAGE_Phage_Gifsy_2_NC_010393(4) | 55.23% |
|  | NODE_46_length_20078_cov_14.707414 | | | | | | | |
|  | 5 | 18.7Kb | incomplete | 30 | 32 | 1240-19959 | PHAGE_Escher_HK639_NC_016158(9) | 52.08% |

|  | Intact (score > 90) |
| --- | --- |
|  | Questionable (score 70-90) |
|  | Incomplete (score < 70) |
