# Supplementary material for: Whole genome sequencing of Klebsiella pneumoniae clinical isolates sequence type 627 isolated from Egyptian patients
Source: PLoS One. 2022 Mar 23;17(3):e0265884. doi: 10.1371/journal.pone.0265884 (PMC8942217; doi:10.1371/journal.pone.0265884)
Supplement: S7 Table — (DOCX) [file pone.0265884.s007.docx]

**S7 Table: SNPs across the core genome of the four isolates belonged to ST 627**

| **Isolate** | **SNP** | **MNP** | **Complex** | **Ins** | **Del** | **Total** |
| --- | --- | --- | --- | --- | --- | --- |
| **K04** | 8786 | 65 | 841 | 49 | 42 | 9783 |
| **K69** | 8825 | 79 | 836 | 47 | 45 | 9832 |
| **K75** | 8806 | 74 | 837 | 48 | 45 | 9810 |
| **K90** | 8775 | 77 | 823 | 45 | 42 | 9762 |
|  |  |  |  |  |  |  |
|  |  |  |  |  |  |  |
| **SNP** | **Single Nucleotide Polymorphism** | | | | |  |
| **MNP** | **Multiple Nuclotide Polymorphism** | | | | | |
| **Ins** | **Insertion** | |  |  |  |  |
| **Del** | **Deletion** | |  |  |  |  |
| **Complex** | **Combination of SNP/MNP** | | | |  |  |
