# Supplementary material for: Whole genome sequencing of Klebsiella pneumoniae clinical isolates sequence type 627 isolated from Egyptian patients
Source: PLoS One. 2022 Mar 23;17(3):e0265884. doi: 10.1371/journal.pone.0265884 (PMC8942217; doi:10.1371/journal.pone.0265884)
Supplement: S8 Table — (DOCX) [file pone.0265884.s008.docx]

**S8 Table: The assessment of the assembled files was carried out by QUAST**

| **Isolate** | **K04** | **K69** | **K75** | **K90** |
| --- | --- | --- | --- | --- |
| **# contigs** | 103 | 93 | 85 | 108 |
| **Largest contig** | 297667 | 510078 | 510078 | 413050 |
| **Total length** | 5486822 | 5486621 | 5485448 | 5538683 |
| **N50** | 151051 | 163013 | 169288 | 119941 |
| **N75** | 73775 | 80853 | 84306 | 70033 |
| **L50** | 15 | 13 | 13 | 14 |
| **L75** | 29 | 26 | 25 | 30 |
