# Supplementary figures and images for: Whole genome sequencing of Klebsiella pneumoniae clinical isolates sequence type 627 isolated from Egyptian patients
Source: PLoS One. 2022 Mar 23;17(3):e0265884. doi: 10.1371/journal.pone.0265884 (PMC8942217; doi:10.1371/journal.pone.0265884)

**S1 Fig: Phylogenetic analysis of the core genome for the four isolates belonged to ST 627**

**
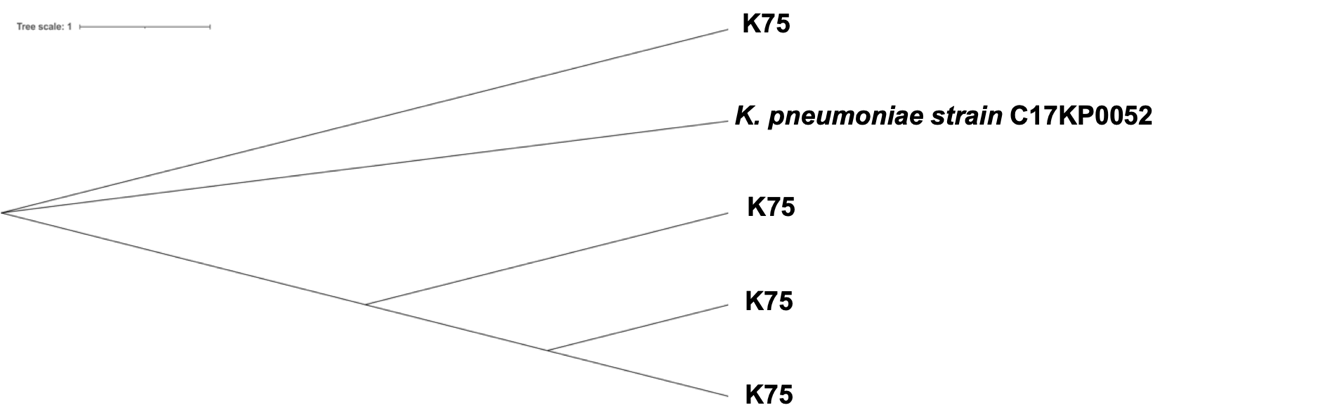
**

Supplement: S1 Fig — (DOCX) [file pone.0265884.s009.docx]
